# Vascular Aging Markers and Blood Biomarkers of Neurodegeneration

**DOI:** 10.1002/alz.095771

**Published:** 2025-01-09

**Authors:** Vanessa A. Guzman, Maliyah Panton, Salwa Rahman, Antonio J Spagnolo Allende, Mitchell S.V. Elkind, Tatjana Rundek, Randolph S. Marshall, Stephanie Cosentino, Jennifer J. Manly, Jose Gutierrez

**Affiliations:** ^1^ Icahn School of Medicine at Mount Sinai, New York, NY USA; ^2^ Columbia University, New York, NY USA; ^3^ Albert Einstein School of Medicine, Bronx, NY USA; ^4^ American Heart Association, Dallas, TX USA; ^5^ Vagelos College of Physicians and Surgeons, Columbia University, New York, NY USA; ^6^ Department of Epidemiology, Mailman School of Public Health, Columbia University, New York, NY USA; ^7^ University of Miami Miller School of Medicine, Miami, FL USA; ^8^ Columbia University Irving Medical Center, New York, NY USA

## Abstract

**Background:**

Age‐related changes in the systemic and cerebral vasculature adversely affect brain health and may contribute to neurodegeneration. However, the relationship between markers of systemic (arterial stiffness) and cerebral (flow pulsatility) vascular aging with neurodegeneration remains unclear. This study aimed to evaluate the associations of arterial stiffness and flow pulsatiility with blood biomarkers of neurodegeneration.

**Method:**

A subset of 80 participants (Mage = 81+7, 63% female, 78% Latinx) from the Northern Manhattan Study completed carotid ultrasound, transcranial doppler (TCD) ultrasonography, and blood sampling. Arterial stiffness was used as a proxy for systemic vascular aging and measured using pulse wave velocity (PWV) obtained from the Vicorder device or carotid doppler. Middle cerebral artery pulsatility index (MCA PI) was used as a proxy for cerebral vascular aging and measured bilaterally using TCD. Blood biomarkers were derived using Simoa Neurology 4‐Plex E kit for: Aβ40, Aβ42, Ptau181, GFAP, and NfL. The ratio of Aβ42 to Aβ40 (Aβ42/Aβ40) was also calculated and included as an outcome measure. We examined the associations of aortic stiffness, common carotid stiffness, internal carotid stiffness, and MCA PI with each blood biomarker, with and without adjustment for age and sex.

**Result:**

MCA PI was associated with increased concentration of Aβ40 (βstandardized = .26, p = .03), p‐tau181 (βstandardized = .31, p = .01), GFAP (βstandardized = .36, p = .002), and NfL (βstandardized = .33, p = .01) but not with Aβ42 (βstandardized = .18, p = .13) or Aβ42/Aβ40 ratio (βstandardized = ‐.05, p = .54). In adjusted models, only the association between MCA PI and ptau181 remained significant (βstandardized = .31, p = .03). There was no association between arterial stiffness measures and blood biomarkers.

**Conclusion:**

Elevated pulsatility in the middle cerebral arteries was associated with increased concentration of blood biomarkers of neurodegeneration; however, associations were attenuated for all blood biomarkers except for p‐tau181 after adjusting for age and sex. These preliminary findings suggest surrogate markers of cerebral vascular aging may be more robust predictors of neurodegeneration. While statistical power was limited by small sample size, future research examining these relationships in larger samples is warranted.

